# *Membranes* 2022 Best Paper Awards

**DOI:** 10.3390/membranes12080756

**Published:** 2022-07-31

**Authors:** Membranes Editorial Office

**Affiliations:** MDPI, St. Alban-Anlage 66, 4052 Basel, Switzerland

*Membranes* is instituting the Best Paper Awards to recognize outstanding papers published in the journal. We are now pleased to announce the winners of the “*Membranes* 2022 Best Paper Awards”.

Papers published in 2020 were preselected by the *Membranes* Editorial Office on the basis of the number of citations and downloads from the website. The winners from the nominations were determined by an award committee and the Editor-in-Chief, together with the Editorial Office. The following two top-voted papers, in no particular order, have won the *Membranes* 2022 Best Paper Awards:

## 1. One Review

Membrane Technologies in Wastewater Treatment: A Review

**Figure 1:**
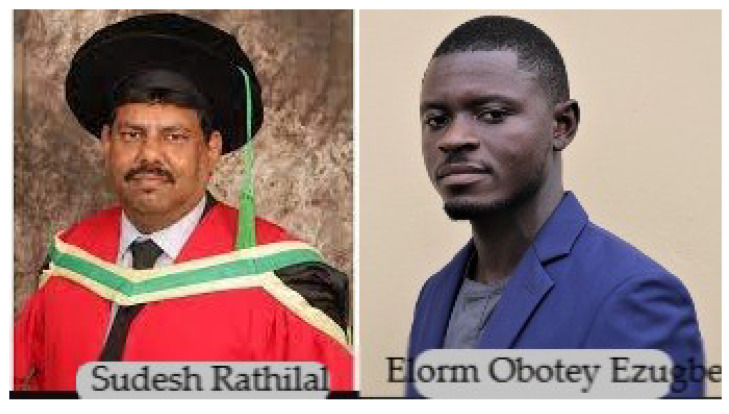
Sudesh Rathilal (left) and Elorm Obotey Ezugbe (right).

By Elorm Obotey Ezugbe and Sudesh Rathilal (Figure 1)

*Membranes* 2020, 10(5), 89; https://doi.org/10.3390/membranes10050089

Available online: https://www.mdpi.com/2077-0375/10/5/89.

Synopsis of the paper by the authors:

In the face of water shortages, the world seeks to explore all available options in reducing the over exploitation of limited freshwater resources. One of the surest available water resources is wastewater. As the population grows, industrial, agricultural, and domestic activities increase accordingly in order to cater for the voluminous needs of humans. These activities produce large volumes of wastewater from which water can be reclaimed to serve many purposes. Over the years, conventional wastewater treatment processes have succeeded to some extent in treating effluents for discharge purposes. However, improvements in wastewater treatment processes are necessary in order to make treated wastewater re-usable for industrial, agricultural, and domestic purposes. Membrane technology has emerged as a favorite choice for reclaiming water from different wastewater streams for re-use. This review looks at the trending membrane technologies in wastewater treatment, and their advantages and disadvantages. It also discusses membrane fouling, membrane cleaning, and membrane modules. Finally, recommendations for future research pertaining to the application of membrane technology in wastewater treatment are made [1].

## 2. One Research Article

Development of Hydrophilic PVDF Membrane Using Vapour Induced Phase Separation Method for Produced Water Treatment

**Figure 2:**
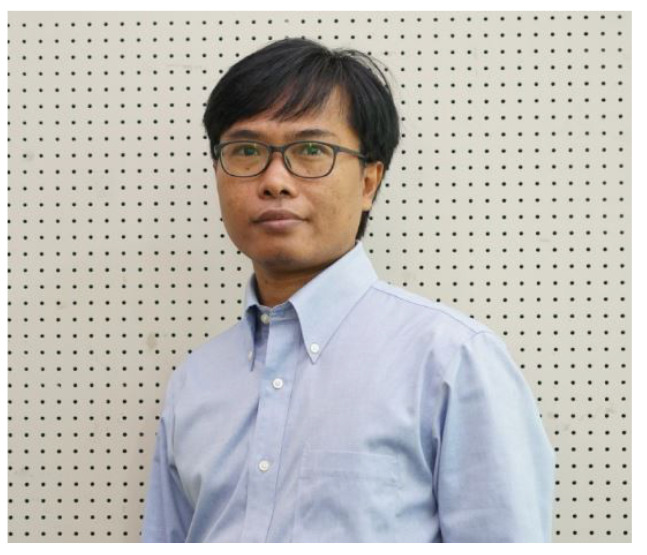
Muhammad Roil Bilad.

By Dr. Muhammad Roil Bilad, Ms. Normi Izati Mat Nawi, Mr. Min Chean Ho, Dr. Norazanita Shamsuddin, Dr. Thanitporn Narkkun, Dr. Kajornsak Faungnawakij and Dr. Asim Laeeq Khan (Figure 2).

*Membranes* 2020, 10(6), 121; https://doi.org/10.3390/membranes10060121

Available online: https://www.mdpi.com/2077-0375/10/6/121

Synopsis of the paper by the authors:

During the production of oil and gas, a large amount of oily wastewater is generated, which would pollute the environment if discharged without proper treatment. As one of the most promising treatment options, membrane material used for oily wastewater treatment should possess desirable properties of high hydraulic performance combined with high membrane fouling resistance. This project employs the vapor-induced phase separation (VIPS) technique to develop a hydrophilic polyvinylidene fluoride (PVDF) membrane with polyethylene glycol (PEG) as an additive for produced water treatment. Results show that thanks to its slow nonsolvent intake, the VIPS method hinders additive leaching during the cast film immersion. The results also reveal that the exposure of the film to the open air before immersion greatly influences the structure of the developed membranes. By extending the exposure time from 0 to 30 min, the membrane morphology changes from typical asymmetric with large macrovoids to the macrovoid-free porous symmetric membrane with a granular structure, which corresponds to 35% increment of steady-state permeability to 189 L·m^−2^h^−1^bar^−1^, while maintaining >90% of oil rejection. It was also found that more PEG content resides in the membrane matrix when the exposure time is extended, contributes to the elevation of surface hydrophilicity, which improves the membrane antifouling properties. Overall results demonstrate the potential of VIPS method for the fabrication of hydrophilic PVDF membrane by helping to preserve hydrophilic additive in the membrane matrices [2].

These two papers represent valuable contributions to *Membranes*. We warmly congratulate both teams on their accomplishments and wish them continued success.

*Membranes* 2022 Best Paper Awards Committee,

*Membranes* Editorial Board.
